# Genetic factors in esophageal atresia, tracheo-esophageal fistula and the VACTERL association: Roles for *FOXF1* and the 16q24.1 FOX transcription factor gene cluster, and review of the literature

**DOI:** 10.1016/j.ejmg.2009.10.001

**Published:** 2010-01

**Authors:** Charles Shaw-Smith

**Affiliations:** a Wellcome Trust Sanger Institute, Hinxton, Cambridge CB10 1SA, UK; b Institute of Child Health, 30, Guilford Street, London WC1N 1EH, UK

**Keywords:** Esophageal atresia, Tracheo-esophageal fistula, VACTERL association, 16q24.1 microdeletion, FOXF1, Sonic hedgehog

## Abstract

Esophageal atresia with/without tracheo-esophageal fistula is a relatively common malformation, occurring in around 1 in 3500 births. In around half of cases, additional malformations are present, forming either a syndrome of known genetic aetiology, or a recognised association, of which the VACTERL association (Vertebral anomalies, Anal atresia, Cardiac malformations, Tracheo-Esophageal fistula, Renal and Limb malformations) is the most recognised. Recently, microdeletions of the FOX gene cluster at 16q24.1, comprising four genes, *FOXF1*, *MTHFSD*, *FOXC2* and *FOXL1*, were reported to cause a phenotype resembling VACTERL association, with vertebral anomalies, gastro-intestinal atresias (esophageal, duodenal and anal), congenital heart malformations, and urinary tract malformations, as well as a rare lethal developmental anomaly of the lung, alveolar capillary dysplasia. This article reviews these new data alongside other genetic causes of syndromic esophageal atresia, and also highlights information from relevant mouse models, particularly those for genes in the Sonic Hedgehog pathway.

## 1 Introduction

Esophageal atresia with or without tracheo-esophageal fistula is a relatively common malformation, occurring in around 1 in 3500 births [50]. In around half of cases, additional malformations are present, forming either a syndrome of known genetic aetiology, of which CHARGE syndrome [OMIM 214800], Feingold syndrome [OMIM 164280] and AEG syndrome [OMIM 206900] are examples; or a recognised association, of which the VACTERL association [OMIM 192350] is the best recognised. Esophageal atresia and the VACTERL association have been the subject of several recent reviews [5,16,50]; however, new data concerning the FOX transcription factor gene cluster at 16q24.1 [52] have provided additional insights, making another look at genetic aetiologies of esophageal atresia and the VACTERL association worthwhile.

A significant factor which has expedited this research is the application of high-resolution array-based methods to patients with congenital malformations [25,49]. Chromosomal deletions in humans result in haploinsufficiency and may therefore disrupt the normal function of any dosage-sensitive genes which they harbour. Genes involved in critical developmental processes, such as transcription factors and signalling molecules, often prove to be dosage sensitive. Examples in humans include SOX2 [OMIM 184429] in eye and foregut development, and N-MYC [OMIM 164840] and, most recently, members of the FOX transcription factor gene cluster at 16q24.1 in foregut and lung development [52].

The application of array-based comparative genomic hybridization (array-CGH) to the detection of haploinsufficiency in humans has therefore proved to be very useful for the identification, amongst other things, of developmentally critical, dosage-sensitive, genes. This technique is now fully integrated into clinical cytogenetics laboratories [2], and in the future may well supersede analysis of G-banded metaphase cells by light microscopy. It has the ability to detect copy number loss (and gain) with extremely high resolution, far higher than was previously achievable in a routine clinical cytogenetics laboratory only a few years ago. This has had two related, beneficial effects: the first is that more pathogenic deletions are being identified; the second is that the deletions are much smaller, and so contain orders of magnitude fewer candidate genes.

Some of the results from the studies using human subjects have been confirmed in other model systems. For example, the efforts of mouse developmental biologists have been hugely valuable in characterizing gene function in developmental processes by targeted mutagenesis of candidate genes in mice. In the field of foregut and lung development, as in many other fields, work on signalling molecules and transcription factors in a wide range of pathways is gradually building up a picture of key events in morphogenesis of the foregut, lung and other organ systems [6,33,44]. This work offers a panel of candidate genes to human geneticists as they analyze the loci which emerge from array data.

In this review, I focus initially on phenotypes associated with microdeletions at 16q24.1, and the role of the individual genes in the FOX transcription factor cluster. I then go on to place these new data in the context of other single gene disorders associated with syndromic esophageal atresia. The contribution of mouse models to our understanding is discussed, followed by a section focussing on the role of Sonic hedgehog and genes in that pathway.

## 2 FOXF1 microdeletions at 16q24.1

Recently it was shown that microdeletions encompassing the FOX cluster at 16q24.1 result in alveolar capillary dysplasia together with a broad spectrum of additional malformations [52]. Alveolar capillary dysplasia with misalignment of pulmonary veins (ACD/MPV) is a rare lethal disorder associated chiefly with failure of development of the intrinsic pulmonary vasculature [[52] and references therein]. There is minimal response to respiratory supportive measures and death usually occurs within the first month of life. In around 80% of cases, additional malformations are present, and the elucidation of the genetic pathology at 16q24.1 in a significant proportion of these cases has enabled some interesting genotype–phenotype correlations to be made. Patients with deletions spanning the entire FOX cluster (*FOXF1*, *MTHFSD*, *FOXC2* and *FOXL1)* at 16q24.1 (these are hereinafter referred to as ‘whole cluster deletions’) have, in addition to ACD/MPV, esophageal atresia, tracheo-esophageal fistula, and other gastro-intestinal tract atresias (duodenal and anal atresia). Congenital heart defects, urinary tract malformations, vertebral or axial malformations and single umbilical artery are also present, making the resemblance to the VACTERL association a compelling one. This raises the possibility that some cases of 16q24.1 microdeletion may be misdiagnosed as VACTERL association, and the accompanying diagnosis of ACD/MPV overlooked. The diagnosis of ACD/MPV can be made only by histology, either at post mortem or by examination of lung biopsy tissue; and even then may be missed, as it is rare and requires some expertise on the part of the pathologist to make it.

Of the four genes in the 16q24.1 FOX cluster, two, *FOXF1* [OMIM 601089] and *FOXC2* [OMIM 602402] have now been reasonably well studied both in humans and the mouse; the remaining two, *MTHFSD* and *FOXL1* [OMIM 603252] have been less well studied. Mutations in *FOXC2* cause lymphoedema–distichiasis syndrome [OMIM 153400]. A comparison of the phenotypes due to *FOXF1* and *FOXC2* mutations in humans, and to *Foxf1* and *Foxc2* inactivation in mouse is shown in Table 1, together with the phenotypes resulting from deletions of the whole FOX cluster in humans. For some organ systems, clear interpretation and correlation for the two genes in human and mouse is possible. For example, in the vertebral/axial system, *FOXC2* mutations, whole cluster deletions, and *Foxc2* knockout mice all result in vertebral/axial malformations [3,11,19,52]; neither humans with *FOXF1* mutations nor *Foxf1* knockout mice appear to manifest these. Similarly, *FOXF1* and *Foxf1*, but not *FOXC2* or *Foxc2*, are clearly associated with abnormalities of intrinsic pulmonary vasculature and lung lobation [23,28,34] while inactivation of *FOXC2* and *Foxc2*, but not *FOXF1* or *Foxf1*, is associated with cleft palate [3,19]. Mutations in both *FOXF1* and *FOXC2*, as well as whole cluster deletions, result in urinary tract malformations [3,52] but there is no reference to this organ system in the literature on *Foxf1* and *Foxc2* mouse mutants.

In the cardiovascular system and gastro-intestinal tract, these correlations are not as simple. *FOXC2* is clearly implicated in cardiovascular malformations, albeit with low penetrance: tetralogy of Fallot in mutation cases [3] and interrupted aortic arch in whole cluster deletion cases [52], correlating well with mouse mutants, which have aortic arch malformations [19]. However, one of the human *FOXF1* mutation cases had a partial AV canal defect [52]; and no such malformations have been reported in *Foxf1* mutant mice.

In the GI tract, *FOXF1* mutations appear consistently to cause intestinal malrotation, as well as a collection of other GI tract anomalies: duodenal stenosis, congenital short bowel, and annular pancreas, but no gastro-intestinal atresia [52]. This contrasts with heterozygous *Foxf1* null mice, which have esophageal atresia and tracheo-esophageal fistula on selected genetic background (CD1), but in which neither intestinal malrotation, nor indeed any other GI tract anomaly, have been reported [35]. In contrast again, humans with whole cluster deletions have not only EA/TEF but also duodenal and anal atresia [52]. No GI tract malformations have been reported in humans or mice with *FOXC2*/*Foxc2* mutations. The possible implications of these observations, and their relationship to the Sonic Hedgehog pathway, are discussed further below.

**Fig. 1:**
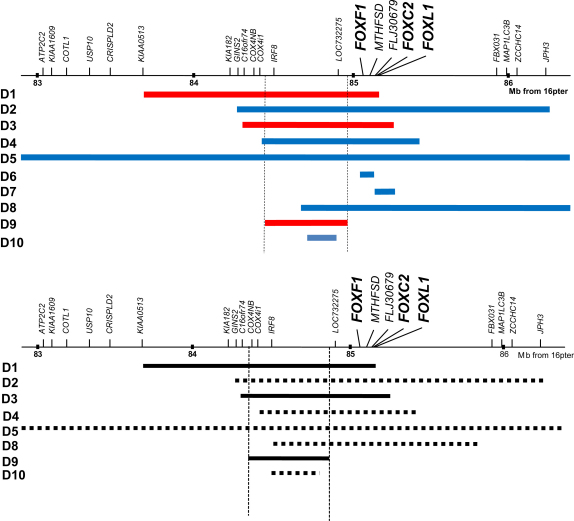
Microdeletions at 16q24.1 in patients with gastro-intestinal atresias Three patients in the recent report of microdeletions at 16q24.1 presented with gastro-intestinal atresias: D1, D3 and D9. One of these, D9, is a deletion upstream of the FOX transcription factor cluster, suggesting a ‘critical interval’ for gastro-intestinal atresias at this locus, that does not span the FOX cluster itself. More deletion cases are needed in order to confirm or refute these preliminary ideas.

Not only whole cluster deletions, but also deletions upstream of the 16q24.1 FOX cluster, are associated with an abnormal phenotype. Such deletions reproduce the alveolar capillary dysplasia phenotype (cases D9 and D10, Fig. 1) and are also associated, in one case, with anal atresia (case D9, Fig. 1), suggesting that the ‘critical interval’ for GI atresias may not actually include the FOX cluster itself (Fig. 1). Future studies will focus on refining this interval by the ascertainment of additional deletion cases.

Mutations in *MTHFSD* and *FOXL1* have not been described in humans. Targeted disruption of *Foxl1* in mice is associated with hyperplasia of the gastric mucosa, abnormal crypt architecture and epithelial cell positioning in the small intestine and retarded growth [32,54]; its role in humans will be unclear until mutations or single gene deletions of *FOXL1* are described in humans.

Comparison of the phenotypes of patients with *FOXF1*, *FOXC2* and whole cluster deletions leads to a hypothesis that the phenotype in patients with whole cluster deletions results from the contiguous deletion of *FOXF1* and *FOXC2* with perhaps additional effects from the other genes or elements in the cluster. This model appears to hold for vertebral, cardiac and renal malformations, but not in the GI tract, where malformations seen in *FOXF1* mutation cases are different from those seen in whole cluster deletion patients. This apparent puzzle is discussed further in the section on the Sonic Hedgehog pathway, below.

Aside from ACD/MPV, there are some phenotypic differences between 16q24.1 microdeletion syndrome and VACTERL association. Hypoplastic left heart syndrome has not previously been reported as a cardiac manifestation of VACTERL association. Limb malformations, particularly of the thumb and radius, are a part of the VACTERL association but do not appear to be present in patients with 16q24.1 microdeletions, while other malformations not part of VACTERL do occur, such as cleft lip and palate in one patient [52].

## 3 Syndromic esophageal atresia/tracheo-esophageal fistula as a single gene disorder

Four disorders in which EA/TEF feature in a significant number of cases, and for which the genetic defect has been elucidated, have been described: these are Feingold syndrome [OMIM 164280], CHARGE syndrome [OMIM 214800], AEG syndrome [OMIM 206900] and VACTERL-H syndrome [OMIM 276950] (see Table 2 and references therein). For the purposes of comparison, the 16q24.1 locus is added to this list in Table 2, accepting the fact that the genetic mechanism in this disorder has not been fully elucidated, and that it may be a contiguous gene deletion syndrome.

There are some striking similarities between the five disorders, as well as some differences. Vertebral and urinary tract malformations occur in all five. All except AEG syndrome feature cardiovascular malformations, with aortic arch abnormalities featuring prominently, and likewise, anal atresia or stenosis occur in these four. GI tract malformations are very similar in Feingold syndrome and 16q24.1 microdeletions, with EA/TEF, duodenal atresia, anal atresia, and annular pancreas occurring in both. There is least concordance for limb malformations, which are of different types in the three conditions in which they occur, VACTERL-H, Feingold and Charge syndromes. There is clearly a danger in overstating the similarities between these diverse conditions, especially as the column labelled ‘other’ contains many anomalies not otherwise classified in the table, but nonetheless, similarities both across and within organ systems, such as those between Feingold syndrome and 16q24.1 microdeletion syndrome, are striking and hint at similarities in pathogenesis.

Esophageal atresia/tracheo-esophageal fistula occur occasionally in other single gene disorders. These include Opitz B/GGG syndrome [OMIM 300000], due to mutations in *MID1* [OMIM 300552] at Xp22.3 [42], in which laryngeal malformations (cleft, diastema) are more usual; McKusick–Kaufman syndrome [OMIM 236700], which is allelic with Bardet–Biedl syndrome [BBS-6, OMIM 209900] and due to mutations in MKKS [OMIM 604896] [51,53], a gene related to members of the chaperonin family; and Oculo-Auriculo-Vertebral Spectrum [OAVS, OMIM 164210], for which epigenetic dysregulation of BPAX1 has been proposed as an aetiological mechanism [14]. Putative single gene or sporadic disorders featuring esophageal atresia for which there is as yet no confirmed genetic aetiology include Fryns syndrome [OMIM 229850] [1], and congenital microgastria–limb reduction complex [OMIM 156810] [31], discussed further below. Finally, there are several reports of a syndrome of multiple gastro-intestinal atresias, including esophageal atresia/tracheo-esophageal fistula, with gall bladder agenesis and neonatal diabetes mellitus, associated in some but not all cases with pancreatic anomalies [8]. Parental consanguinity and sibling recurrence point to autosomal recessive inheritance, but there are as yet no mapping data.

## 4 Mouse models featuring esophageal atresia/tracheo-esophageal fistula

A list of mouse models featuring esophageal atresia or tracheo-esophageal fistula is provided in Table 3. Prominent on this list are members of the Sonic Hedgehog pathway, including Sonic Hedgehog itself. *Shh*−/− mice have an extensive set of malformations which overlaps very significantly not only with the VACTERL association, but also with the set of malformations seen in patients with microdeletions at 16q24.1 (excluding ACD/MPV) [9,52]. Mutations in *SHH* in humans cause holoprosencephaly [HPE3, OMIM 142945] [40] and microphthalmia [OMIM 611638] [48]. Mutations have not been reported in patients with malformations of the gastro-intestinal tract. The *GLI* family of transcription factors are downstream effectors of *SHH* and in humans, mutations in members of this gene family result in syndromes well known to clinical geneticists. Greig cephalopolysyndactyly [GCPS, OMIM 175700] and Pallister–Hall [PHS, OMIM 146510] syndromes are caused by mutations in *GLI3* [20], while mutations in *GLI2* are associated with holoprosencephaly [47]. None of these syndromes manifests foregut malformations, although anal atresia is a component of Pallister–Hall syndrome; however, mice with inactivation of both *Gli2* and *Gli3* do have foregut malformations (summarized in Table 3), and again it seems worthwhile to search for mutations in *GLI* family members in patients with syndromic esophageal atresia.

Mice with *Noggin*−/− mutations have a wide range of malformations including esophageal atresia and tracheo-esophageal fistula [27,43]. Tantalizingly, a clear link between the NOGGIN locus at 17q22 and esophageal atresia has been established in humans [36], with most recently, the critical interval narrowed to a 5.9 Mb region encompassing NOGGIN [41]. The phenotype in these deletion cases has been tentatively delineated to include deafness and skeletal malformations (symphalangism, joint contractures) as well as EA/TEF, and so mutations in NOGGIN could usefully be sought in deletion-negative patients with this phenotype.

Finally, a mouse model was published recently in which mutation of the proprotein convertase enzyme *Pcsk5* was shown to be associated with malformations in the VACTERL spectrum, including defects of tracheo-oesophageal septation [60]. Convincing mutations in this gene in humans with VACTERL association have yet to be identified.

## 5 Dissection of Shh and Foxf1 function in model organisms

**Fig. 2:**
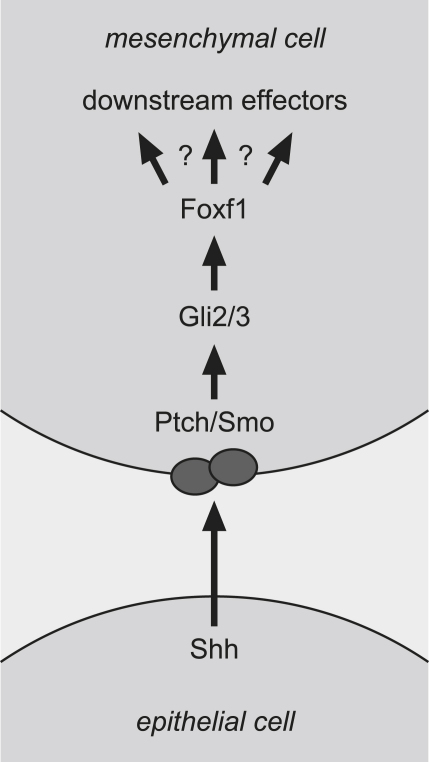
Epithelial–mesenchymal interactions mediated by the Sonic Hedgehog pathway in the gut. Sonic hedgehog effects signalling between the epithelial and mesenchymal cell in the gastro-intestinal tract, and acts indirectly upon Foxf1 via its receptors, Smo and Ptch, and Gli transcription factors. The effects of Foxf1 are mediated within the mesenchyme, but there is currently much uncertainty surrounding the identity of the downstream effectors of Foxf1. Shh, Sonic Hedgehog; Ptch, Patched; Smo, Smoothened.

Key emerging players in the pathogenesis of esophageal atresia, tracheo-esophageal fistula and the VACTERL association are members of the Sonic Hedgehog pathway, and of the FOX transcription factor gene cluster at 16q24.1. Studies in model organisms including *Xenopus* and mouse have demonstrated key roles for Sonic Hedgehog itself, and for *Foxf1*, in mediating mesoderm–endoderm cross talk during very early development of the gut tube. A critical early step is the subdivision of lateral plate mesoderm into its somatic and visceral components, and evidence from *Xenopus* [56] and mouse [34] assigns a key role to *Foxf1* in this process. Subsequently, the dorsal mesentery, from which the early gut tube is suspended from the dorsal abdominal wall, and the splanchnic mesoderm lining the gut tube, develop from the visceral component of the lateral plate mesoderm. Signalling between the visceral mesoderm, which develops into smooth muscle and other specialized tissues in the gut wall, and the gut endoderm, which provides the epithelial cell lining of the gastro-intestinal tract, is critical for correct development of the gut [37]. There is compelling evidence that *Foxf1* is a downstream effector of the Sonic hedgehog pathway in this process, both in the lung and in the gut. In the lung, ectopic epithelial expression of *Shh* activates *Foxf1* expression [35]; in *Shh*−/− mice, Foxf1 mRNA is absent from its usual sites of expression in mesenchyme of trachea and oesophagus, and lungs, although midgut and hindgut do retain some expression [35]. Similarly, in the intestine, but not the stomach, of both *Gli2* and *Gli3* null mice, *Foxf1* levels are reduced [32]. The *Foxf1* promoter, as well as that of *Foxl1*, contains binding sites for *Gli* factors which mediate transcriptional activation [32]. It does appear that expression of *Foxf1* is not exclusively controlled by the Sonic Hedgehog pathway, as evidenced by its retained expression in the intestine of *Shh*−/− mice [35], but clearly there is some regulation of *Foxf1* by this pathway, and the close link between the two is evident in the phenotypic similarities of mouse mutants and human patients with *FOXF1* mutations and deletions [52]. More detailed analysis of GI tract malformations in *Shh*−/− mice has revealed more extensive abnormalities: intestinal malrotation, annular pancreas, duodenal stenosis, abnormal gut innervation, and intestinal transformation of stomach [46]. Strikingly, the first three malformations on this list are also seen in patients with *FOXF1* mutations [52]. *Sonic Hedgehog* therefore appears to provide a link between the set of malformations seen in *FOXF1* mutation cases and the set seen in whole cluster deletion cases: the malformations that occur are all seen in *Shh*−/− mice. The relationship between *Shh* and *Foxf1* is further explored in Fig. 2.

In mice, the phenotypic consequences of *Shh*, *Gli* transcription factor and *Foxf1* inactivation are shown in Table 3; the resemblances to VACTERL association have already been commented upon. How do we relate these observations from model organisms to relevant human phenotypes? For *SHH*, human mutations so far described have resulted in holoprosencephaly and microphthalmia, but these have so far been mis-sense mutations or in-frame deletions. Where are patients with inactivating mutations and deletions of this gene? One possibility is that these mutations are lethal at an early embryonic stage; a second possibility is that investigators have not looked in the right phenotypic group. One reported phenotype is the Microgastria–Limb Reduction complex [OMIM 156810], which features terminal transverse limb defects similar to those seen in *Shh*−/− mice, intestinal malrotation, esophageal and anal atresia, megacolon, abnormal lung lobation, heart defects, renal and CNS anomalies. This shows some similarities to the phenotype of *Shh*−/− mutant mice, but, against this, three reported cases of this condition have been in discordant twin pairs, suggesting that the aetiology may be related to the twinning process [31].

Concerning *FOXF1* and the 16q24.1 FOX gene cluster, the interesting question remains: why does *FOXF1* inactivation result in intestinal malrotation, but deletion of the entire FOX cluster result in gastro-intestinal atresias, whereas *Foxf1* inactivation in mice results in esophageal atresia? Future studies will focus on narrowing the ‘critical interval’ responsible for occurrence of GI atresias (esophageal, duodenal and anal) in patients with microdeletions at 16q24.1. It will be interesting to determine whether *FOXL1*, which also has a role in intestinal development, contributes to this phenotype in humans.

## 6 Chromosomal imbalances

A comprehensive review of chromosomal anomalies in esophageal atresia/tracheo-esophageal fistula was published recently [12], and in this section, attention is drawn to just a few of these. Trisomies for chromosomes 18 and 21 have been associated with EA/TEF; interestingly more cases have been associated with trisomy 18 despite the fact that it is much rarer than trisomy 21 [50]. The locus perhaps closest to revealing a new causative gene is 17q22, harbouring NOG, discussed above [36]. A link between chromosomal deletions at chromosome 13q32, and VACTERL-type malformations has previously been postulated [58], although in fact deletions within the region 13q22-13qter have been associated with a very broad spectrum of malformations [24,45] and the suggested link with VACTERL association probably reflects ascertainment bias, at least to some extent. The zinc finger transcription factor ZIC2 [OMIM 603073] appears to be responsible for the major CNS malformations occurring in patients with deletions at this locus [45]. Just one case with esophageal atresia has been reported [58]; nonetheless, there does appear to be a definite link with anal atresia and peno-scrotal transposition mapping to 13q33, and the critical region for this malformation appears to exclude ZIC2 [15]. Esophageal atresia/tracheo-esophageal fistula are rare associations in patients with deletions at chromosome 22q11.2 [10]. Finally, microdeletions spanning the FOX transcription factor gene cluster at chromosome 16q24.1 should now be added to this list [52], but further genotype–phenotype studies are required in order to clarify precisely which genes or sequence elements are responsible.

## 7 Concluding remarks

The recent delineation of the role of the 16q24.1 locus in the aetiology of severe developmental malformations illustrates very clearly the role that high-resolution microarrays can play in improving our understanding of developmental disorders. All of the deletions reported in affected patients are below the level of cytogenetic resolution, but very easily detectable using microarrays. Recent work on chromosomal anomalies in EA/TEF suggests that there are other haploinsufficient susceptibility loci for these and related malformations, notably at 13q and 17q, and quite naturally as a result of clinical studies, our knowledge will grow. There is also a clear utility in research studies focussed on a particular malformations: our own study, the Genetics of Oesophageal Atresia http://www.ich.ucl.ac.uk/ich/academicunits/MMU/CustomMenu_01 now has high-resolution array data on close to 100 patients with syndromic esophageal atresia, with some potentially interesting loci. These data will be published in due course.

A complementary approach is the sequencing of genes in the same cohort of patients, looking for mutations both in known (*N-MYC*, *SOX2* and others) and in candidate (*SHH*, *NOG* and others) genes. Sequencing can be carried out on a large scale on whole-genome amplified DNA, as has recently been described for the whole X chromosome [55]. Ultimately, the goal of these studies is to complete the ‘cytogenetic map’ for esophageal atresia and tracheo-esophageal fistula. This work is now well under way, and will be useful not only for its own sake but also as a complement to studies of non-genetic factors in EA/TEF, which have to date been restricted mostly to large-scale epidemiological studies.

## Figures and Tables

**Table 1:** Phenotypes associated with haploinsufficiency of FOXF1 and FOXC2 in humans and of *Foxf1*, *Foxc2* and *Shh* in mouse.

Gene/locus	Vertebrae	Anal	Cardiac	Tracheo-Esophageal	Renal	Limb	Respiratory	Cranio-facial	Other	References
*FOXF1*	–	–	AV canal defect, PDA	–	Hydronephrosis, hydroureter, dilated bladder	–	ACD/MPV, abnormal lung lobation	–	Intestinal malrotation, annular pancreas, duodenal stenosis, congenital short bowel,	[52]
*Foxf1*	–	–	–	EA/TEF	–	–	ACD-like but no MPV. Lung lobation anomalies	–	Gall bladder small with abnormal smooth muscle	[23,28,35]
*FOXC2*	Scoliosis, rib fusion	–	Tetralogy of Fallot, VSD, PDA	–	Duplex kidney, pyelonephritis, urinary tract infections	–	–	Cleft palate	Lymphoedema–distichiasis, varicose veins, ptosis	[3,11]
*Foxc2*	Split neural arches, absent spinous processes, rib fusions	–	Interrupted aortic arch, aortic coarctation, aortic atresia, VSD	–	–	–	–	Cleft palate, hypoplastic/fused middle ear bones	–	[19]
Deletions of whole FOX cluster	Butterfly vertebra, rib fusions	Anal atresia	HLHS, Tetralogy of Fallot, Interrupted aortic arch, PDA	EA/TEF	Hydronephrosis, renal pelvicaliectasis	–	ACD/MPV, pneumothorax pulmonary lymphangiectasia	Cleft lip, cleft palate, brachycephaly	Single umbilical artery	[52]
*Shh*	Agenesis of axial skeleton	Anal atresia	Abnormalities of cardiac looping	EA/TEF	Renal agenesis	Limb truncation defects	Lung lobation	Cyclopia	Intestinal malrotation, annular pancreas, duodenal stenosis, abnormal gut innervation, intestinal transformation of stomach	[9,29,46]

AV=atrioventricular, VSD=ventricular septal defect, PDA=patent ductus arteriosus, HLHS=hypoplastic left heart syndrome, ACD/MPV=alveolar capillary dysplasia/misalignment of pulmonary veins.

**Table 2:** Syndromic esophageal atresia due to single gene disorders in humans, with microdeletion at 16q24.1 for comparison.

Syndrome/gene	Vertebrae	Anal atresia/other GI malformations	Cardiac	Tracheo-Esophageal	Renal	Limb	Other	References
Feingold syndrome *N-MYC*	Fused cervical vertebrae, absent sacral vertebrae, absent rib pairs	Anal atresia, also duodenal atresia or stenosis, annular pancreas	Tricuspid stenosis/atresia, interrupted aortic arch, VSD, PDA	EA, TEF	Hydronephrosis, cystic dysplasia, dilatation of renal pelvis, small kidneys	Short middle phalanges of 2nd and 5th digits, hypoplastic thumbs, toe syndactyly (2nd and 3rd, or 4th and 5th toes)	Microcephaly, learning difficulties, deafness, short stature, asplenia or polysplenia	[7]

VACTERL-hydrocephalus *FANCB*	Lumbar spina bifida occulta, cervical vertebral defects	Anal atresia	Aortic coarctation, ASD, VSD,	EA, TEF	Unilateral renal agenesis, renal dysplasia	Bilateral radial agenesis, absent thumbs	Fanconi anaemia, hydrocephalus, Arnold–Chiari malformation; cleft palate, incomplete lung lobation	[18,30,38]

Charge syndrome *CHD7*	Vertebral body anomalies, kyphoscoliosis	Anal stenosis	HLHS, Tetralogy of Fallot, DORV, AVSD, right-sided descending aorta, Shone's complex, ASD, VSD, PDA	EA, TEF	Renal agenesis, horseshoe kidney, vesico-ureteric reflux, renal cysts	Monodactyly, tibial aplasia, bifid femora	Coloboma of eye, structural anomalies of external ear, deafness, agenesis of semi-circular canals, choanal atresia, cleft lip, cleft palate, cryptorchidism, micropenis, hydrocephalus, corpus callosum agenesis, seizures, learning difficulties	[21,57]

AEG syndrome *SOX2*	Hemi-vertebrae, butterfly vertebrae, fused ribs, absent ribs, extra ribs	–	–	EA, TEF	Hypoplastic kidneys, duplex kidneys	–	Anophthalmia, microphthalmia, hypospadias, cryptorchidism	[59]

16q24.1 microdeletion encompassing *FOXF1, MTHFSD*, *FOXC2* and *FOXL1*	Butterfly vertebra, rib fusions	Anal atresia, posterior placement of anus, duodenal atresia, annular pancreas	HLHS, Tetralogy of Fallot, Interrupted aortic arch, PDA	EA, TEF	Hydronephrosis, renal pelvicaliectasis	–	ACD/MPV, cleft lip, cleft palate, brachycephaly, single umbilical artery	[52]

AVSD=atrioventricular septal defect, ASD=atrial septal defect, VSD=ventricular septal defect, PDA=patent ductus arteriosus, HLHS=hypoplastic left heart syndrome, ACD/MPV=alveolar capillary dysplasia/misalignment of pulmonary veins, DORV=double outlet right ventricle.

**Table 3:** Knockout mouse models featuring esophageal atresia and/or tracheo-esophageal fistula, with phenotype associated with inactivation of counterpart human gene for comparison.

*Mouse gene*	Lung/foregut phenotype	Phenotype (other)	Reference	Human gene	Locus	Mutations	Lung/foregut phenotype	Phenotype (other)	Reference
*Shh*	Esophageal atresia, tracheo-esophageal fistula, lung anomalies	Abnormalities of CNS; cyclopia; distal limb truncation; abnormalities of axial skeleton, renal agenesis, abnormalities of heart looping, intestinal malrotation, annular pancreas, duodenal stenosis, intestinal transformation of the stomach, abnormal gut innervation, imperforate anus	[9,29,46]	*SHH*	7q36	YES	NR	Holoprosencephaly [HPE3, OMIM 142945], microphthalmia [OMIM 611638]	[40,48]

*Foxf1*	Esophageal atresia, tracheo-esophageal fistula, lung lobe fusion defects, pulmonary vascular defects	Axial skeletal defects	[22,35]	*FOXF1*	16q24.1	YES	Alveolar capillary dysplasia	Intestinal malrotation, atrio-ventricular canal defect, renal malformations	[52]

*Gli2*	*Gli2*−/− mice are normal. Gli2−/− Gli3+/− mice have esophageal atresia tracheo-esophageal fistula. *Gli2*−/− *Gli3*−/− mice have absent oesophagus, trachea and lungs	NR	[39]	*GLI2*	2q14	YES	NR	Holoprosencephaly	[47]

*Gli3*	See *Gli2* entry	*Gli3*−/− mice are allelic with Xt. They have synpolydactyly and brain malformations.	[39]	*GLI3*	7p13	YES	NR	Greig cephalopolysyndactyly syndrome [GCPS, OMIM 175700]; Pallister–Hall syndrome [PHS, OMIM 146510] (imperforate anus, polydactyly, hypopituitarism, hypothalamic hamartoblastoma)	[20]

*Noggin*	Esophageal atresia, tracheo-esophageal fistula	Failure of closure of neural tube, exencephaly, wide, club-shaped limbs, shortened, abnormal body axis, lethality at birth	[27,43]	*NOGGIN*	17q22	YES	NR	Proximal symphalangism with multiple synostoses; stapes ankylosis with broad thumbs and toes; brachydactyly type B2	[4,17,26]

*Sox2*	Esophageal atresia, tracheo-esophageal fistula	Neurodegeneration, impaired neurogenesis	[13,44]	*SOX2*	3q26.3	YES	Esophageal atresia	Anophthalmia, genitourinary malformations	[59]

NR = not recorded.
